# Management of Placenta Previa in a Jehovah's Witness Patient

**DOI:** 10.1002/ccr3.9706

**Published:** 2024-12-06

**Authors:** Aariya Srinivasan, Jibran Ikram, Sabry Ayad

**Affiliations:** ^1^ Department of Anesthesiology Research Cleveland Clinic Foundation Cleveland Ohio USA; ^2^ Cleveland Clinic Foundation Cleveland Ohio USA

**Keywords:** hysterectomy, Jehovah's Witness, posterior placenta previa, postpartum hemorrhage (PPH), uterine artery embolization (UAE)

## Abstract

Placenta previa is the partial or complete covering of the internal os of cervix. It is one of the major risk factors for postpartum hemorrhage (PPH), maternal and neonatal morbidity and mortality. A 36‐year‐old G3P2 Jehovah's Witness female with a gestational age of 36 weeks 6 days and past medical history of chronic hypertension, T2DM, asthma, and past obstetric history of two cesarean sections, large for gestational age babies, and postpartum hemorrhage due to uterine atony, underwent an elective repeat low transverse cesarean section in view of posterior complete placenta previa, complicated by massive postpartum hemorrhage. The patient was immediately transported to the interventional radiology (IR) for uterine artery embolization (UAE) after unsuccessful treatment attempts with uterotonics and JADA system (vacuum induced uterine tamponade). In view of rapidly progressing bleeding, the decision was made for an urgent hysterectomy. Management of postpartum hemorrhage in a Jehovah's Witness patient is particularly complex as these patients refuse administration of blood products and involves medical, ethical, and legal implications.

**Summary**

- Effective management of postpartum hemorrhage (PPH) in Jehovah's Witness patients requires a comprehensive, multidisciplinary approach that respects their refusal of blood products.
- Early identification of risk factors, timely intervention with uterotonics, and innovative techniques like uterine balloon tamponade are critical. In cases of uncontrolled hemorrhage, prompt transfer to interventional radiology and consideration of surgical options, including hysterectomy, are essential to ensure maternal safety.
- Collaboration among obstetricians, anesthesiologists, and interventional radiologists is vital to optimize outcomes in this complex clinical scenario.

## 1 Introduction

Postpartum hemorrhage (PPH) is one of the leading causes of pregnancy‐related deaths in the world. PPH is defined as a cumulative blood loss of more than 1000 mL or signs and symptoms of hypovolemia present within 24 h of the birthing process [1]. Management of PPH becomes particularly challenging in Jehovah's Witness patients who refuse blood transfusions. Early diagnosis or identification of risk factors for postpartum hemorrhage plays a major role in pregnant patients for whom transfusion is not an option. Management of PPH should be carried out meticulously in a stepwise fashion and definitive life saving measures should often be carried out earlier than usual. Here, we present a case of a 36‐year‐old pregnant female with posterior placenta previa, complicated by PPH after repeat low transverse cesarean section, highlighting the multidisciplinary management approach and a brief review of literature.

## 2 Case History and Examination

A 36‐year‐old Gravida 3 Para 2, Jehovah's Witness female patient with a gestational age of 36 weeks 6 days was admitted for a scheduled repeat Low transverse cesarean section in view of posterior placenta previa. Her past medical history included chronic hypertension, T2DM, and asthma. She has a past obstetric history of two cesarean sections, large for gestational age babies, and PPH due to uterine atony. A comprehensive pre‐partum planning and management was done for this patient. With placenta previa and history of two cesarean sections and after a discussion with maternal and fetal medicine, MRI was done to rule out PAS disorders. The possibility of hysterectomy was discussed with the patient considering the high risk. Plan for cell saver was discussed along with cryoprecipitate and albumin in the context of blood product refusal.

## 3 Investigations and Diagnosis

**FIGURE 1:**
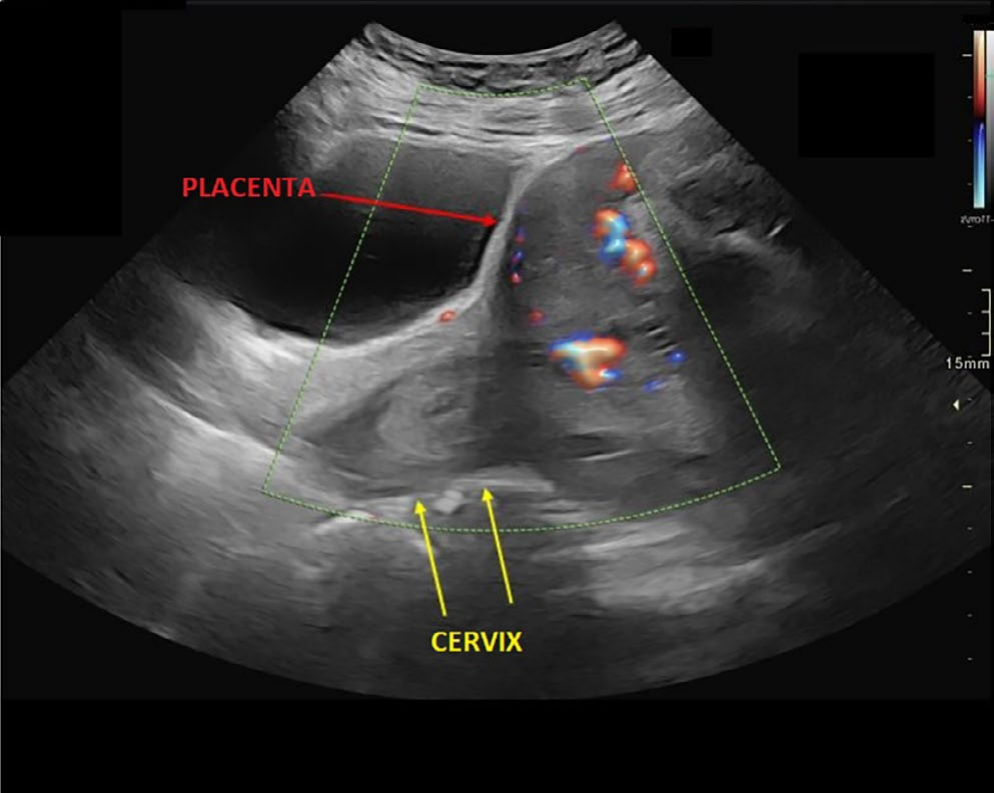
Ultrasound image showing the placenta previa and the cervix.

**FIGURE 2:**
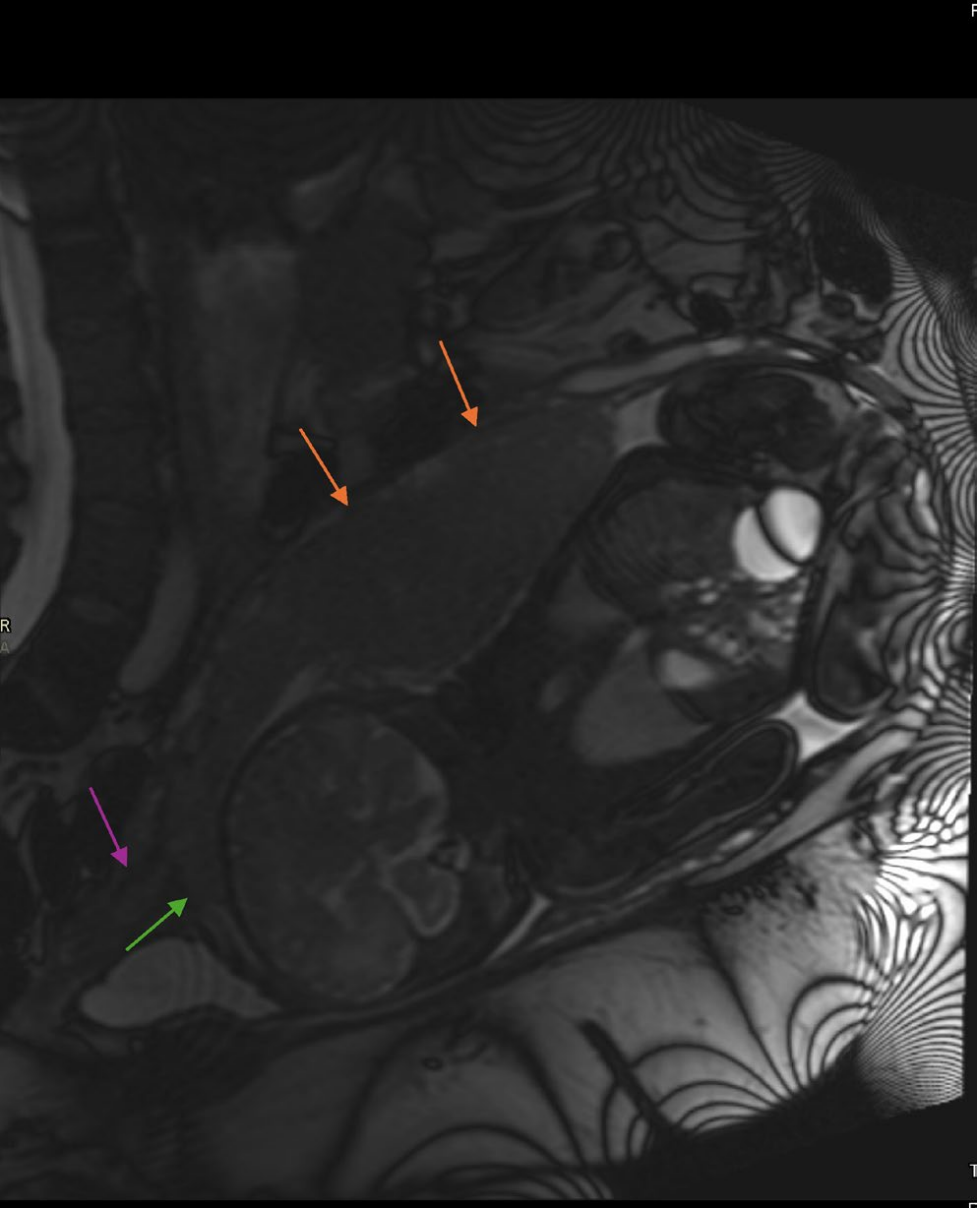
Placenta lying posteriorly—orange arrow; cervix—purple arrow; complete placenta previa with placenta over internal os without appreciable myometrial thinning or loss of retroplacental clear space. There was no hypervascularity of the uterovesical space and no lacunae—green arrow.

Trans‐vaginal ultrasound done at 36 weeks of gestation showed complete placenta previa with placenta over internal os and extending anteriorly without appreciable myometrial thinning or loss of retroplacental clear space (Figure 1). There was no hypervascularity of the uterovesical space and no lacunae. Even though placenta accreta spectrum disease was excluded by MRI Pelvis (Figure 2), histopathological report showed signs of delayed villous maturation, retroplacental hemorrhage, and high‐grade fetal vascular malperfusion with nonocclusive thrombi in chorionic plate and stem villous vessels. Adherent basal plate myometrial fibers with intervening decidua suggest possible placental accreta spectrum, warranting clinical correlation.

## 4 Management

The baby was delivered through a low transverse cesarean section under spinal anesthesia with bupivacaine–dextrose 0.75% injection. She received a total of 1100 mL of intravenous fluids. The patient was stable, and the birth weight of the baby was 2.956 kg with 1‐min Apgar of 9 and 5‐min Apgar of 9. Her delivery was complicated by PPH with a cumulative blood loss of about 1400 mL. According to her blood transfusion consent form, she would only accept minor blood fractions including albumin, clotting factors and cryoprecipitate. The patient was treated with 200 mg of misoprostol, 10‐units bolus oxytocin twice, and 1000 mg of tranexamic acid. The posterior bed of the uterus was oversewed with figure of eight stitches. There was no bleeding at this point and vital signs were stable. Later in the post anesthesia care unit, patient had bleeding from the placental site despite firm fundus for which a vacuum induced uterine tamponade, JADA was placed through transvaginal route into the uterine cavity gently by compressing the uterine loop. Once in place, the cervical seal placement was confirmed and inflated with a volume of 120 mL of sterile saline. The vacuum source was turned on at a pressure of 80 mmHg. The bleeding initially stopped and vital signs were stable. The plan for Uterine artery embolization and possible hysterectomy was discussed with the patient if Vacuum assisted uterine tamponade was not successful in stopping the placental site bleeding. Despite the use of JADA system (vacuum‐induced uterine tamponade) and uterotonics, patient continued to bleed around the balloon. Patient continued to decline PRBCs and platelets despite discussing the risks associated with PPH. The patient was taken to interventional radiology for uterine artery embolization and the bleeding progressively increased during the procedure. Fibrinogen was 178 mg/dL at this point. The patient's vital signs remained stable without pressure support throughout the procedure. After consulting the blood bank physician, consent for 1‐unit FFP was obtained after discussion with the family and continued cryoprecipitate and albumin. The total estimated blood loss at this point was 3500 mL. Maternal fetal medicine was notified regarding the need for possible hysterectomy. Abdominal aortogram, right internal iliac, and right uterine artery angiograms showed multiple bleeding sites within the uterus and cervix. Partial embolization of the right uterine artery was performed using Gelfoam slurries and one‐third vial of 900–1200 μm Embozenes. In view of persistent bleeding within the uterus and concern for progression to disseminated intravascular coagulation the procedure was stopped, and patient was scheduled for urgent hysterectomy. The PT, APTT, and INR were within normal limits at this time. She was then transferred to the operation room for hysterectomy and bilateral salpingectomy. The patient was hemodynamically stable and on low‐dose phenylephrine for pressure support and was transferred intubated to the ICU. Patient remained intubated. Patient's postoperative Hgb decreased to 4.6 from 11.6 g/dL preoperatively. Total estimated blood loss at this point was around 4.5 L. She received a total of 4.5 L of crystalloids, 2 units of cryoprecipitate, 1 unit of Fresh frozen plasma, 1.5 L of albumin, in addition to 1000 mg of tranexamic acid and 80 cc of cell saver intra operatively.

The patient was extubated on postoperative day 1. Hematology was consulted in view of Hemoglobin nadir of 4.6 g/dL, platelet nadir of 144 k/μL and fibrinogen nadir of 178 mg/dL. She has been treated with weekly darbepoetin, IV iron and supplemented with Vitamin B12 and Folic acid. She was found to have ischemic changes on EKG that was done the morning after hysterectomy, compared to the one done prior to the cesarean section and hysterectomy, which was normal. There were abnormalities related to profound anemia with nonspecific intraventricular conduction delay and acute anterior infarct. Troponin at this point was 10 ng/L and CK MB was 1.2%. The patient was monitored on telemetry continuously after consulting cardiology and was treated conservatively for profound anemia. There was no evidence of acute kidney injury with good urine output.

## 5 Outcome and Follow‐Up

The patient was discharged on postoperative day 4 with a hemoglobin (Hgb) level of 5.8 g/dL. Treatment with erythrocyte stimulating agents, iron, vitamin B12, and folic acid was continued. Over the following weeks, her hemoglobin showed a significant improvement, increasing by 50% to 10.5 g/dL during a routine postpartum office visit approximately 2 weeks and to 12.3 g/dL at around 6 weeks after surgery. The patient and the baby are doing well, with no additional complications or issues reported.

## 6 Discussion

Obstetric hemorrhage is considered as one of the most dangerous complications of pregnancy and childbirth. Primary causes of PPH can include uterine atony, genital tract lacerations, retained placenta, uterine inversion, abnormal placentation, and coagulation disorders. Secondary causes include retained products of conception, infection, sub‐involution of the placental site, inherited coagulation deficits, and placenta previa. Of all these causes, placenta previa and history of PPH in previous pregnancies are significant high‐risk factors for massive PPH. Hypertensive disorders and anemia also proved to be notable risk factors among others [2, 3]. Regardless of the mode of delivery, a blood loss of more than or equal to 1000 mL is considered as PPH [2, 3]. Risk stratification by health services and optimization of prenatal, childbirth, and postnatal care can help in prevention of PPH. One of the main modes of prevention of PPH is “Rule of three” which is slow infusion of 3 units of oxytocin at 3‐min intervals for up to three doses, followed by maintenance dose intravenous infusion (15 units in 500 mL of 0.9% NS at the rate of 100 mL/h) [3].

Placenta previa occurs as a complication in about 0.3%–0.5% of pregnancies. It is accepted as clinically significant if the lower edge of placenta lies within ≤ 2 cm from the internal os. Previous cesarean sections increased maternal age and parity have shown to be associated high risk factors for placenta previa [4]. This patient was a representation of PPH from posterior complete placenta previa extending anteriorly and most likely contributing factors could have been history of previous cesarean sections, history of PPH, history of dilatation and curettage and advanced maternal age.

Patients with combined previous cesarean section and placenta previa were more likely to have adverse maternal outcomes especially the need for hysterectomy [5, 6]. Placenta previa can lead to life threatening hemorrhages in any stage of pregnancy including antepartum, intrapartum, and postpartum period. Among the cases of placenta previa, the most common type associated with PPH is complete placenta previa and anterior localization of the placenta [7]. Although less frequent, PPH can still occur in women with incomplete placenta previa which are located on the posterior wall of the uterus [8].

Abnormal placentation, including placenta previa and placental accreta spectrum (PAS) disorders, is known to be the major risk factor for postpartum hemorrhage (PPH). Therefore, in Jehovah's Witness patients with a history of previous cesarean section, it is recommended to perform a detailed ultrasound evaluation early in pregnancy (at around 6–8 weeks) to assess for the risk of “scar pregnancy” or abnormal placentation. Additionally, it is important to note that ultrasound diagnostic challenges can arise in cases of multiple pregnancies, succenturiate placentas, or rare conditions such as multiple pregnancies with a complete hydatidiform mole alongside a normal fetus. In such cases, a dislocated second placenta may complicate the evaluation and increase the difficulty in diagnosing PAS disorders [9].

Patients with placenta previa are usually managed by cesarean section, uterotonic agents, hemostatic sutures and blood transfusions if required. Despite such measures, the bleeding cannot be controlled in some patients and peripartum hysterectomy becomes the most viable option to save the patient's life. The relative risk of the need for hysterectomy in patients with placenta previa is about 30–40 [4]. In this patient timely action of performing hysterectomy was vital for the successful outcome.

The management of placenta previa leading to PPH becomes particularly challenging when the patient is a Jehovah's Witness and refuses to accept blood products. Pharmacological agents like oxytocin, misoprostol and carboprost are some of the uterotonics commonly used in such patients. Tranexamic acid is also widely used as it inhibits conversion of plasminogen to plasmin which is known to cause fibrinolysis by competitively inhibiting the activation of plasminogen. There have been few positive outcomes [10], but little evidence for the use of DDAVP in the management of PPH and is mostly used in patients with disturbed platelet function, which could have been caused by drugs, acidemia, or hypothermia [11]. Uterine Balloon tamponade and compression sutures are known to effectively manage PPH in the absence of blood transfusion. The intrauterine vacuum‐induced hemorrhage control device (JADA system) is a novel technology in the non‐surgical invasive treatment of PPH. It is one of the most studied vacuum aspiration device. Its success rate is 88.2% in cesarean section PPH [3]. It was used in our patient in view of failure of control of bleeding by pharmacological measures.

Aggressive fluid resuscitation helps maintain intravascular volume and helps the patient to be hemodynamically stable. Vasopressors are used in case of heavy bleeding and hypotension for pressure support and plays a major role in Jehovah's Witness patients. Obstetricians should also discuss the possibility of mechanical ventilation and admission to intensive care unit to reduce the oxygen consumption [12].

Iron therapy and erythropoiesis stimulating agents are used for replenishing iron stores and stimulate red blood cell production. High‐dose erythropoietin stimulating agents was used to treat severe PPH in Jehovah's Witness patients successfully. It led to an improvement in this patient's postoperative hemoglobin to about 50%, when used in combination with iron therapy [13, 14].

## 7 Conclusion

Optimal management of PPH in a Jehovah's Witness patient requires a complex and multidisciplinary approach at various points of care. Risk stratification and preventive measures including administration of oxytocin by “Rule of three” play a major role in the management of PPH in cesarean sections and to reduce adverse maternal outcomes. However, a careful consideration of all treatment modalities and timely action by using uterotonics, tranexamic acid, cryoprecipitate, cell saver, JADA system (vacuum‐induced uterine tamponade), uterine artery embolization, and hysterectomy as a last resort with erythropoietin and IV Iron postoperatively is crucial to successfully manage PPH in Jehovah's Witness patients.

## Author Contributions

**Aariya Srinivasan:** conceptualization, writing – original draft, writing – review and editing. **Jibran Ikram:** conceptualization, writing – original draft, writing – review and editing. **Sabry Ayad:** conceptualization, supervision, validation, writing – review and editing.

## Consent

Written informed consent was acquired from the patient whose case details are written in the study, to publish this report in accordance with the journal's patient consent policy.

## Conflicts of Interest

The authors declare no conflicts of interest.

## Data Availability

The data supporting this report's findings are available on request from the corresponding author. These data are not publicly available due to privacy or ethical restrictions.

## References

[1] American College of Obstetricians and Gynecologists , “Practice Bulletin No. 183: Postpartum Hemorrhage,” Obstetrics and Gynecology 130, no. 4 (2017): e168–e186, https://pubmed.ncbi.nlm.nih.gov/28937571/. 28937571 10.1097/AOG.0000000000002351

[2] K. C. Wormer , R. T. Jamil , and S. B. Bryant , “Postpartum Hemorrhage,” In StatPearls [Internet] (Treasure Island, FL: StatPearls Publishing, 2023), https://www.ncbi.nlm.nih.gov/books/NBK499988/. 29763164

[3] Á. L. L. Alves , A. A. Francisco , G. C. Osanan , and L. B. Vieira , “Postpartum Hemorrhage: Prevention, Diagnosis and Non‐Surgical Management: Number 5–November 2020,” Revista Brasileira de Ginecologia e Obstetrícia 42, no. 11 (2020): 776–784, 10.1055/s-0040-1721882. 33254276 PMC10416182

[4] E. Giambattista , M. W. Ossola , S. F. Duiella , et al., “Predicting Factors for Emergency Peripartum Hysterectomy in Women With Placenta Previa,” Archives of Gynecology and Obstetrics 285, no. 4 (2012): 901–906, 10.1007/s00404-011-2074-8. 21898078

[5] A. H. Shaamash , M. H. AlQasem , A. A. Mahfouz , D. S. Al Ghamdi , and M. A. Eskandar , “Major Placenta Previa Among Patients With and Without Previous Cesarean Section: Maternal Characteristics, Outcomes and Risk Factors,” European Journal of Obstetrics, Gynecology, and Reproductive Biology 296 (2024): 280–285, 10.1016/j.ejogrb.2024.03.012. 38493552

[6] L. J. King , A. Dhanya Mackeen , C. Nordberg , and M. J. Paglia , “Maternal Risk Factors Associated With Persistent Placenta Previa,” Placenta 99 (2020): 189–192, 10.1016/j.placenta.2020.08.004. 32854040

[7] H. J. Lee , Y. J. Lee , E. H. Ahn , et al., “Risk Factors for Massive Postpartum Bleeding in Pregnancies in Which Incomplete Placenta Previa Are Located on the Posterior Uterine Wall,” Obstetrics & Gynecology Science 60, no. 6 (2017): 520, 10.5468/ogs.2017.60.6.520. 29184859 PMC5694725

[8] L. Tuzovic , “Complete Versus Incomplete Placenta Previa and Obstetric Outcome,” International Journal of Gynaecology and Obstetrics 93, no. 2 (2006): 110–117, https://pubmed.ncbi.nlm.nih.gov/16563394/. 16563394 10.1016/j.ijgo.2006.02.006

[9] V. Giorgione , P. Cavoretto , G. Cormio , et al., “Prenatal Diagnosis of Twin Pregnancies with Complete Hydatiform Mole and Coexistent Normal Fetus: A Series of 13 Cases,” Gynecologic and Obstetric Investigation 82, no. 4 (2017): 404–409. 27522447 10.1159/000448139

[10] S. A. Kozek‐Langenecker , A. Afshari , P. Albaladejo , et al., “Management of Severe Perioperative Bleeding: Guidelines From the European Society of Anaesthesiology,” European Journal of Anaesthesiology 30 (2013): 270–382. 23656742 10.1097/EJA.0b013e32835f4d5b

[11] D. E. Trigg , I. Stergiotou , P. Peitsidis , and R. A. Kadir , “A Systematic Review: The Use of Desmopressin for Treatment and Prophylaxis of Bleeding Disorders in Pregnancy,” Haemophilia 18 (2012): 25–33. 21624012 10.1111/j.1365-2516.2011.02573.x

[12] T.‐H. Kim , H.‐H. Lee , and J.‐M. Kim , “Recommendations for Postpartum Hemorrhage in Women Who Decline Blood Transfusion,” Acta Obstetricia et Gynecologica Scandinavica 94 (2015): 786, 10.1111/aogs.12630. 25753728

[13] G. Schälte , H. Janz , J. Busse , V. Jovanovic , R. Rossaint , and R. Kuhlen , “Life‐Threatening Postoperative Blood Loss in a Jehovah's Witness, Treated With High‐Dose Erythropoietin,” British Journal of Anaesthesia 94, no. 4 (2005): 442–444, https://academic.oup.com/bja/article/94/4/442/292441. 15653706 10.1093/bja/aei068

[14] A. M. Ball and P. S. Winstead , “Recombinant Human Erythropoietin Therapy in Critically Ill Jehovah's Witnesses,” Pharmacotherapy 28, no. 11 (2008): 1383–1390, 10.1592/phco.28.11.1383. 18956998

